# Examination of how food environment and psychological factors interact in their relationship with dietary behaviours: test of a cross-sectional model

**DOI:** 10.1186/s12966-019-0772-y

**Published:** 2019-01-30

**Authors:** Christina Vogel, Gavin Abbott, Georgia Ntani, Mary Barker, Cyrus Cooper, Graham Moon, Kylie Ball, Janis Baird

**Affiliations:** 1 Medical Research Council Lifecourse Epidemiology Unit, University of Southampton, Southampton General Hospital Tremona Road, Southampton, SO16 6YD UK; 2 Institute for Physical Activity and Nutrition Research, School of Exercise and Nutrition Sciences, Deakin University, 221 Burwood Hwy, Burwood, Victoria 3125 Australia; 3 National Institute for Health Research Southampton Biomedical Research Centre, University of Southampton and University Hospital Southampton NHS Foundation Trust, Southampton, SO16 6YD UK; 4 School of Geography and Environmental Science, University of Southampton, University Road, Southampton, SO17 1BJ UK

**Keywords:** Dietary behaviour, Food environment, Psychological resources, Agency, Food affordability, Modelling

## Abstract

**Background:**

To improve population diet environmental strategies have been hailed the panacea because they require little agency or investment of personal resources; this contrasts with conventional strategies that rely on individuals to engage high levels of agency and make deliberate choices. There is an immediate need to improve understanding of the synergy between the psychological and environmental determinants of diet in order to optimise allocation of precious public health resources. This study examined the synergistic and relative association between a number of food environment and psychological factors and the dietary behaviours of a population sample of women with young children.

**Methods:**

Women in Hampshire were recruited from children’s centres and asked about their demographic characteristics, psychological resources, dietary behaviours (food frequency questionnaire) and perceptions of healthy food access and affordability. Three local food environment factors were objectively assessed: i) spatial access to food outlets using activity spaces; ii) healthfulness of the supermarket where women did their main food shop, (based on nine in-store factors including price, placement and promotion on seven healthy and five less healthy foods); iii) nutrition environment of children’s centres visited frequently by the women, assessed via staff-administered questionnaire. A theoretical model linking environmental factors to dietary behaviours, both directly and indirectly through three factors representing individual agency (psychological resources, perceived food affordability, perceived food accessibility), was tested using Structural Equation Modelling.

**Results:**

Complete data were available for 753 women. The environment of women’s main supermarket was indirectly related to their dietary behaviours through psychological resources and perceived food affordability. Shopping at supermarkets classified as having a healthier in-store environment was associated with having greater psychological resources associated with healthy eating (standardised regression weight β = 0.14SD, *p* = 0.03) and fewer food affordability concerns (β = − 0.14SD, *p* = 0.01), which in turn related to healthier dietary behaviours (β = 0.55SD, < 0.001 and β = − 0.15, p = 0.01 respectively). The three food environment factors were not directly associated with dietary behaviour (*p* > 0.3). The overall model fit was good (CFI = 0.91, RMSEA = 0.05 [0.05, 0.06]).

**Conclusions:**

This pathway analysis identified three focal points for intervention and suggests that high-agency interventions targeting individual psychological resources when combined with low-agency supermarket environment interventions may confer greater benefits on dietary behaviours than either intervention alone.

## Background

The limited effectiveness of educational and information campaigns at tackling stubborn public health problems like obesity and poor diet is now widely recognised [1, 2]. It has been proposed that these types of interventions are limited by their requirement for high levels of individual agency, or purposeful enactment of available psychological, cognitive, social and financial resources, to be effective [3, 4]. Alternative low-agency public health interventions, which require little investment of an individual’s resources and involve addressing the broader environmental determinants of health behaviours such as fiscal measures, school food policies or product reformulation, are considered to hold great potential for being effective and wide-reaching [3, 5]. Evidence from two systematic reviews supports the notion that high-agency obesity-prevention interventions, like nutrition information campaigns, produce relatively small effects, particularly among those with the poorest dietary behaviours [6, 7]. However, there is contradictory evidence from another review which found little difference in the influence of obesity-related policies on health inequalities according to their required level of agency [8]. These findings suggest that calls to shift policy and research activity towards either low- or high-agency initiatives to improve health behaviours, like diet, may be oversimplifying the complexity of human behaviour [9, 10].

Contemporary theoretical frameworks, like the socioecological model, emphasise that health behaviours are the product of synergistic action between individual, social, environmental, policy and economic factors [11]. A number of conceptual models also postulate specific pathways of influence, indicating that environmental factors can act directly on diet, and/or indirectly through psychological mediators such as perceived control [12–14]. However, with most research having focused on the direct relationships, few studies have tested potential mediators of the pathway between food environment exposures and diet [15, 16]. There is a clear need for empirical evidence to understand the relative effect of, and synergistic action between, the various psychological and environmental determinants of diet to inform appropriate allocation of precious public health resources [14].

This study applied the widely used conceptual framework, the model of community nutrition environments [12], to test the relative importance of a number of environmental and psychological factors in their association with dietary behaviours among a population sample of women with young children, with good representation of those from disadvantaged backgrounds. All model components were identified from existing evidence of dietary determinants among this population [15, 17–19].

**Fig. 1:**
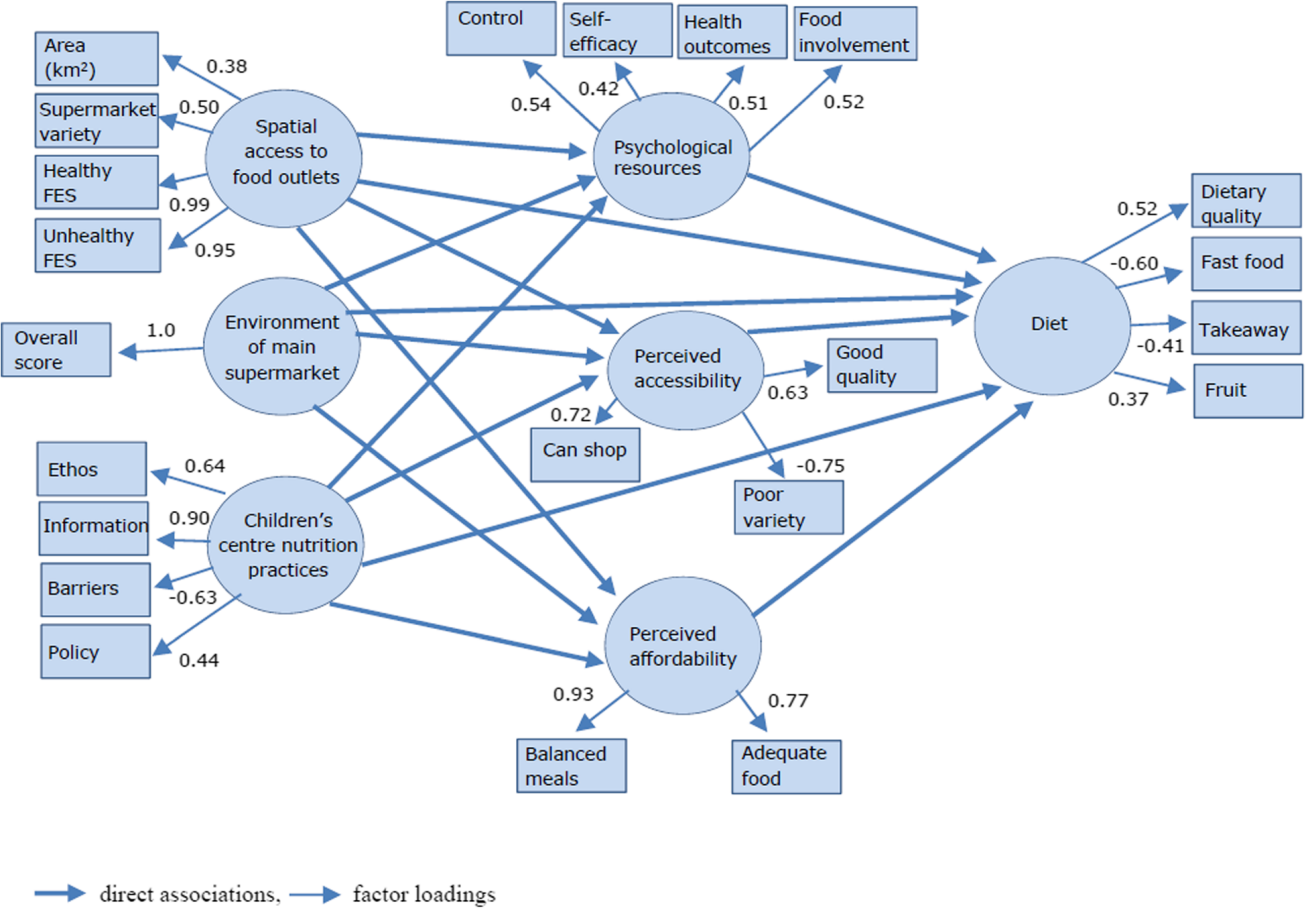
Measurement model showing standardised factor loadings for multiple indicator latent constructs

The model (Fig. 1) posits that dietary behaviours are directly affected by three individual-level psychological factors: i) psychological resources (e.g. perceived control), ii) perceived food affordability, and iii) perceived food accessibility; these factors assume high levels of individual agency. The model also hypothesises that dietary behaviours are directly affected by three food environment factors that require lower levels of agency: i) spatial access to food outlets, ii) in-store environment of main supermarket, and iii) nutrition environment of children’s centres frequently visited. Finally, the model proposes synergistic action where the three food environment factors act indirectly on dietary behaviours, through the psychological factors. These direct and indirect relationships were examined using structural equation modelling (SEM). This technique has a number of advantages over other multivariate techniques including simultaneous assessment of multiple interrelated relationships to enable direct comparison, and improved estimation of relationships by using latent constructs to reduce measurement error [20].

## Methods

### Study design and area

This study was cross-sectional and used participant data from the Southampton Initiative for Health (SIH), a study of women attending Sure Start Children’s Centres in Hampshire, UK [21, 22]. Sure Start Children’s Centres were a UK government initiative introduced to provide health, education and support services for families with young children aged under 5 years [23]. These centres emphasised support for vulnerable families and offered play groups, parenting courses, child health checks and housing or employment services. Healthy eating was a priority issue for Sure Start Children’s Centres with nutrition information, snacks and cooking sessions frequently offered to parents and children using these services [24]. The environmental data originated from observational surveys of food outlets and a cross-sectional telephone survey with children’s centre staff. The study area covered the three council areas of the SIH (Southampton, Gosport and Havant) plus Eastleigh, Fareham and Portsmouth because participants reported food shopping and working in these neighbouring areas. Southampton, Portsmouth and Havant have concentrated areas of high deprivation and are ranked in the most deprived third of the 326 local authorities in England; Gosport, Eastleigh and Fareham are more affluent [25]. More than 98% of the study area was classified as urban. All study procedures, including acquiring written consent from all participants, were conducted according to the Declaration of Helsinki and were approved by the University of Southampton, Faculty of Medicine Ethics Committee (SOMSEC025.09, SOMSEC033.09, SOMSEC037.09, SOMSEC065.10).

### Participants

Participants were women who were pregnant or had a young child and whose home residence and main supermarket were located within the study area. All women were recruited while attending children’s centres located in Southampton, Gosport and Havant. Local Sure Start data indicated that 70% of children aged under 5 years were registered with one of their children’s centres at the time of the study [21]. Detailed information about the 30% not registered was not available, however, children’s centre staff believed that the most advantaged and most disadvantaged families were least likely to engage with their services. A total of 509 participants who had previously completed the phase I SIH survey in 2009 undertook phase II by telephone between December 2010 and May 2011. During the same time period, an additional 412 were recruited in phase II to enhance sample numbers and completed the questionnaire face-to-face. Analysis of differences between the two groups of participants showed that mothers completing only phase II were younger (*p* < 0.001), more likely to have one child (p < 0.001), had lower levels of educational attainment (*p* = 0.04) and lived in more deprived neighbourhoods (*p* = 0.02) than mothers who completed both Phase I and II surveys. The phase II recruitment bolstered the numbers of disadvantaged mothers and by combining both groups the sample in this study had representation from across the socioeconomic spectrum. All participant information was treated as cross-sectional.

Questions were asked about women’s age, number of children, highest educational qualification attained, home postcode, and postcodes of frequently visited locations (main supermarket, workplace, children’s centre, general practitioner and physical activity site). Home postcode was used to determine participants’ level of neighbourhood deprivation according to quintiles of the 2007 English Index of Deprivation income domain [26]. The questionnaire also included items relating to dietary behaviours, psychological resources and perceptions of the local food environment which are described below.

### Dietary outcome construct

Table 1 summarises the four measures used to describe the **dietary outcome latent construct**: dietary quality score, fruit intake, fast-food intake and takeaway food intake. A dietary quality score was calculated for each participant using a 20-item food frequency questionnaire (FFQ) The score has been validated against serum folate, a biomarker of nutritional status [27]. Participants were asked how often in the previous month they consumed each of the 20 foods. Dietary quality scores were calculated by multiplying consumption frequency for each item by corresponding coefficients identified from a principal components analysis and summing the results. Scores were standardised to have a mean of zero and standard deviation (SD) of one. Higher scores represented better dietary quality aligned with the national Department of Health and Social Care’s dietary recommendations in England (The Eatwell Guide) [28], characterised by higher intakes of vegetables and wholegrain bread, and lower intakes of processed meats, crisps and granulated sugar added to cereals, tea or coffee. Fruit intake was assessed separately by a question that asked how often in the previous month fresh fruit was consumed [29]. Fast food and takeaway intake were assessed by asking how often in the past month foods from i) fast food chains and ii) independent takeaway outlets were consumed [30, 31]. Examples of fast food chains and takeaway outlets were provided to facilitate appropriate responses. Exploratory factor analysis showed these four measured variables loaded onto a single dietary latent construct using qualitative assessment and quantitative criteria of eigenvalue greater than one [32] and factor loadings greater than 0.32 [33]. The total variance explained by the dietary latent construct was 23%.

**Table 1:** Summary of measured variables used to determine the dietary, individual and environmental latent constructs

Variable	Measure	Scale	Reliability/Validity
Dietary Outcome			
Dietary quality (SD)	20-item food frequency questionnaire (FFQ)	7-point scale from ‘never’ to ‘more than once a day’	Diet scores from the 20-item FFQ have correlated highly with scores from a 100-item FFQ (r=0.94), and with red blood cell folate (r=0.25) [27]
	Dietary scores calculated by multiplying consumption frequency by coefficients from principal components analysis and summing total	Higher scores represented better dietary quality	
		Scores standardised (mean=0, SD=1)	
Fruit intake	‘How often in the previous month have you consumed fresh fruit?’	7-point scale from ‘never’ to ‘more than once a day’	Scale used in previous research [29]
Fast-food intake	‘How often in the past month foods from fast food chains?’	7-point scale from ‘never’ to ‘more than once a day’	Scale used in previous research [30]
Takeaway intake	‘How often in the past month foods from independent takeaway outlets?’	7-point scale from ‘never’ to ‘more than once a day’	Scale used in previous research [31]
Note: Exploratory factor analysis showed these dietary variables loaded onto a single construct: eigenvalue=1.7, factor loadings ≥0.37, explained 23% variance of diet construct			
Psychological resources			
Perceived control over life	9 item scale	4-point from ‘strongly disagree’ to ‘strongly agree’	Published scale [35]
	Responses summed to create overall score (range 5 to 20)	Higher score indicated greater sense of control	Cronbach’s alpha statistic 0.91
	Example items:		
	- ‘I often have the feeling that I am being treated unfairly’		
	- ‘There are certain things I can do for myself to reduce the risk of cancer’		
Self-efficacy for healthy eating	5 item scale	4-point from ‘strongly disagree’ to ‘strongly agree’	Published scale [36]
	Responses summed to create overall score (range 5 to 20)	Higher score indicated greater competence in overcoming barriers to healthy eating	Cronbach’s alpha statistic 0.89
	Example items:		
	‘I could stick to eating healthy foods even if….’		
	- I have to rethink my whole diet		
	- I have to try a few times before I succeed		
Healthy eating outcome expectancies	6 item score	4-point from ‘strongly disagree’ to ‘strongly agree’	Published scale [37]
	Responses summed to create overall score (range 4 to 24)	Higher score indicated stronger beliefs in good outcomes from eating healthily	Cronbach’s alpha statistic 0.84
	Example items:		
	‘I know if I eat healthy foods...’		
	- I won’t have weight problems		
	- It will be good for my blood pressure		
Food involvement	12-item scale	5-point: ‘strongly disagree’, ‘disagree’ ‘neither agree or disagree’, ‘agree’, ‘strongly agree’	Published scale [38]
	Responses summed to create overall score (negative items reversed, range 5 to 60)	Higher score indicated food-related activities are valued and prioritised in daily life	Cronbach’s alpha statistic 0.67
	Example items:		
	- ‘I don’t think much about food each day’		
	- ‘I enjoy cooking for others and myself’		
Note: Exploratory factor analysis showed these dietary variables loaded onto a single construct: eigenvalue=1.5, factor loadings ≥0.48, explained 11% variance of the 3 individual-level constructs			
Perceived food affordability			
Can’t afford enough food	‘In the last 12 months, the food I bought didn’t last and I didn’t have money to get more’	3-point: ‘never true’, ‘sometimes true’, ‘always true’	Scale used in previous research [39]
Can’t afford balanced meals	‘In the last 12 months, I couldn’t afford to eat balanced meals’	3-point: ‘never true’, ‘sometimes true’, ‘always true’	Scale used in previous research [39]
Note: Exploratory factor analysis showed these variables loaded onto a single construct: eigenvalue=2.0, factor loadings ≥0.75, explained 17% variance of the 3 individual constructs			
Perceived food accessibility			
Can food shop locally	‘In my local neighbourhood (10-15 minute walk), I can do most of my food shopping’	4-point from ‘strongly disagree’ to ‘strongly agree’	Scale used in previous research [40]
Limited variety of fresh fruit and vegetables locally	‘In my local neighbourhood, the variety of fresh fruit and vegetables is limited’	4-point from ‘strongly disagree’ to ‘strongly agree’	Scale used in previous research [40]
Good quality produce locally	‘In my local neighbourhood, the fresh produce is usually of a high quality’	4-point from ‘strongly disagree’ to ‘strongly agree’	Scale used in previous research [40]
Note: Exploratory factor analysis showed these variables loaded onto a single construct: eigenvalue=1.9, factor loadings ≥0.63, explained 17% variance of the 3 individual constructs			
Spatial access to food outlets			
Area (km^2^) of activity space	Total space covered by set of Euclidean 1000m (0.6 mile) buffers around postcode centroid of home and other frequently visited locations including main supermarket, work, GP, Sure Start children’s centre and physical activity location.	Total area of buffers in square kilometres (km^2^)	Similar measure used in previous research [68, 69]
		Overlapping buffers were merged to create one total area	
		Range= 4.0km^2^ to 18.0km^2^	
Supermarket variety	Number of different types of supermarkets according to the categories: premium, large, discount and small	Variety values ranged from zero to four	Similar scale used in previous research [70]
	Different types of supermarkets have been shown to differ in terms of product availability, price and promotion [44]	Higher score showed greater variety of supermarkets within activity space	
Food environment score (FES) – healthy outlets	Scores represented both the density of healthy food outlets and a proxy of the healthfulness of the in-store environment based on healthy food availability	Weightings for healthy outlets (0 to 10):	Scale used in previous research [43]
	Scores were calculated by i) identifying the number of each type of healthy food outlets within activity space, and ii) multiplying the number of each food outlets by a corresponding weighting (0 to 10) describing healthy food availability for that outlet determined from a published Delphi study [43]	8.8: F&V store/ farm shop	
		6.3: premium/large supermarket	
		5.4: butcher	
		5.3: ‘world’ store	
		4.9: small supermarket	
		4.4: sandwich shop	
		4.3: health food shop	
		3.3: discount supermarket	
		0.8: bakery	
		Range= 12 to 445.4	
Food environment score (FES) – unhealthy outlets	Scores represented both the density of unhealthy food outlets and a proxy of the healthfulness of the in-store environment based on unhealthy food availability	Weightings for unhealthy outlets (0 to -10):	Scale used in previous research [43]
	Scores were calculated by i) identifying the number of different types of unhealthy food outlets within activity space, and ii) multiplying the number of each food outlets by a corresponding weighting (0 to -10) describing unhealthy food availability for that outlet determined from a published Delphi study [43]	-1.1: convenience/ petrol stores	
		-1.6: Chinese/ Indian takeaway	
		-5.0: fish & chips/ other takeaways (pizza/kebab)	
		-5.0: newsagents/ confectioners	
		-8.3: fast food outlets	
		Range= -9.3 to -753.3	
Note: Exploratory factor analysis showed these variables loaded onto a single construct: eigenvalue=2.4, factor loadings ≥0.33, explained 55% variance of the spatial access to food outlets construct			
Environment of main supermarket			
Healthfulness score	Information on the number of varieties, price, promotion, shelf placement, and store placement were collected about seven healthy (peppers, tomatoes, lettuce, onions, apples, bananas, wholemeal bread) and five unhealthy products (oven chips, sausages, crisps, granulated sugar, white bread)	Z-scores for each of the nine in-store variables were created by subtracting the summed ratings for unhealthy products from the summed ratings for healthy products and standardizing the result	Reliably distinguishes different types of supermarkets [71]
			Inter-rater reliability kappa>0.73 for all except quality (kappa=0.60) [44]
	Data about type of nutrition information and availability of healthier alternatives were collected for unhealthy products	The z-scores for the nine variables were then summed and divided by 9 to ensure each in-store variable was equally weighted	Cronbach’s alpha statistic 0.86
	The quality of two fruits and four vegetables, and whether or not the fruits could be bought singly, were assessed	Scores standardised (mean=0, SD=1)	
		Higher scores show more healthful supermarket environments	
Children’s centre nutrition practices			
Food policy content	21 item scale	Responses were summed	Adapted from two published scales [45, 46]
	1 item assessed food policy existence	Responses: ‘no’, ‘verbal’, ‘written’, ‘unsure’	Cronbach’s alpha statistic 0.74
	13 items – ‘Does your centre policy include: (examples)	3-point scale: ‘yes’, ‘no’, ‘unsure’	
	- Promoting water intake		
	- Restricting soft drinks		
	items – ‘When does the policy apply?’: (examples)	4-point scale: ‘always’, ‘often’, ‘sometimes’, ‘never’	
	- Food provided for children by your centre		
	- Food provided for parents by parents		
	- Food provided for staff by your centre		
	2 items		
	- ‘How often are parents told about the policy?’	4-point scale: ‘always’, ‘often’, ‘sometimes’, ‘never’	
	- ‘How often to parents bring food against the policy?’	Higher scores represent a detailed, wide reaching food and nutrition policy	
Healthy eating ethos	14 item scale	Responses were summed	Adapted from two published scales [45, 46]
	11 items – ‘How regularly do the following practices occur?’: (examples)	4-point scale: ‘always’, ‘often’, ‘sometimes’, ‘never’	Cronbach’s alpha statistic 0.69
	- Staff join children at the table when food is eaten		
	- Parents consume the same food offered to children		
	- Children encouraged to try new or less favoured foods		
	2 items:		
	- ‘How often are healthy eating activities included in centre sessions?’:		
	- ‘How often do staff discuss healthy eating with parents?’		
	1 item assessed:	4-point scale: ‘always’, ‘often’, ‘sometimes’, ‘never’	
	- ‘How important is healthy eating as one the many issues you help parents with at your centre?’		
		10-point scale from ‘not important’ to ‘very important’	
		Higher scores represent stronger healthy eating ethos at the centre	
Healthy eating information	6 item scale	Responses were summed	Adapted from a published scale [45]
	‘How available are the following resources?’: (examples)	3-point scale: ‘readily available’, ‘available on request’, ‘not available’	Cronbach’s alpha statistic 0.52
	- Leaflets/posters about healthy eating or weight loss for adults		
	- Leaflets/posters about weaning or breastfeeding	Higher scores represent better availability of healthy eating resources	
	- Courses to learn to cook or cook on a budget		
Barriers to promoting healthy eating	7 item scale	Responses were summed	Adapted from a published scale [72]
	‘Please indicate the extent to which you feel the following hinder healthy eating promotion at your centre’: (examples)	3-point scale: ‘major problem’, ‘minor problem’, ‘not a problem’	Cronbach’s alpha statistic 0.73
	- Food regulations	Lower scores represent fewer perceived barriers to promoting healthy eating	
	- Parents don’t believe or trust your advice		
	- Cost of healthy eating		
Note: Exploratory factor analysis showed these variables loaded onto a single construct: eigenvalue=2.3, factor loadings ≥0.49, explained 46% variance of the Sure Start nutrition practices construct			

### Individual-level constructs (agency)

Table 1 summarises the measures used to describe the three latent constructs that represent individual agency (*psychological resources*, *perceived food affordability*, *perceived food accessibility*). These constructs were identified from exploratory factor analysis of measured variables previously theorised and shown to predict dietary behaviours [12, 18, 19, 34]. The total variance explained by the three psychological constructs was 45%. Two measured variables, i) social support for fruit and vegetable purchasing and ii) perceived accessibility of healthy takeaway options in residential neighbourhood, returned very low factor loadings indicating that these variables represent different underlying processes to the other measured variables; they were therefore omitted.

The **psychological resources latent construct** was characterised by four measured variables: i) perceived control over life, ii) self-efficacy for healthy eating, iii) healthy eating outcome expectancies, and iv) food involvement. These variables were measured using published scales with good reliability (Table 1) [35–38]. The **perceived food affordability latent construct** was characterised by two measured variables to assess participants’ food circumstances in the past year. Participants were asked whether they could afford to buy i) enough food and ii) balanced meals [39]. The **perceived food accessibility latent construct** was characterised by three measured variables that assessed participant’s perceptions of i) food accessibility, ii) the variety of fresh fruit and vegetables and iii) the quality of fresh produce within a 10–15 min walk or 5 minute drive from their home [40]. These perceived food environment measures were adapted from those used in previous research (Table 1).

### Environmental constructs

Table 1 summarises the measures used to describe the three environmental latent constructs. Exploratory factor analysis was used to determine the measured variables that characterised the **spatial access to food outlets** latent construct. Four measures of spatial access were included: i) square kilometre of individual activity space ii) variety of supermarkets in activity space, iii) food environment score for healthy outlets in activity space, and iv) food environment score for unhealthy outlets in activity space. The total variance explained by the spatial access to food outlet latent construct was 55%. The methods used to collect the spatial access data and create the individualised activity spaces and food environment scores have been described elsewhere [41]. In brief, individualised activity spaces were produced by creating 1000 m (0.6 mile) buffers around postcode centroids of home and frequently visited locations using ArcGIS [42]. Buffers that overlapped were merged into one space and the total area was calculated (km^2^). Cross-sectional food outlet data were collected during observational ‘ground-truthing’ of the study area between July 2010 and June 2011. A total of 1787 outlets were geocoded to postcode centroid using Geoconvert and ArcGIS (< 3% of locations did not match and Google maps was used to identify a proximal address). Coordinates for 20 types of retail and takeaway food outlets were overlaid onto activity spaces to determine the variety of supermarkets, and derive a healthy and an unhealthy food environment score for each participant (Table 1). The food environment scores (FES) represented spatial access to healthy and unhealthy food outlets respectively, and included weightings to characterise the healthfulness of the in-store environments based on the availability of healthy or unhealthy foods in each outlet type [43].

The **environment of main supermarket** latent construct was described by a composite score representing the healthfulness of the in-store environment of each participant’s main supermarket (where they did most of their food shopping) using published methods [44]. In brief, information about nine in-store factors (variety, price, quality, promotion, shelf placement, store placement, nutrition information, healthier alternatives, and single fruit sale) on 12 foods known to discriminate between better and poorer dietary quality were collected via in-store surveys. Data were collected between July 2010 to June 2011, to correspond with timing of participant interviews, from all supermarkets and convenience stores located in the study area. These data were used to create a standardized healthfulness z-score for each supermarket where women shopped (Table 1). The single composite score was used because conceptually all nine components are considered important indicators of the in-store environment.

Exploratory factor analysis revealed that four measured variables characterised the **children’s centre nutrition practices** latent construct: i) food policy content, ii) healthy eating ethos, iii) availability of healthy eating information, and iv) barriers to promoting healthy eating. The total variance explained by the children’s centre nutrition practices latent construct was 46%. Data were collected via cross-sectional telephone survey, from August to October 2011, with a convenience sample of 86 staff members at 28 children’s centres located in the study area. Responses from staff members of the same centre (*n* = 2–5) were averaged to provide a single response from each centre for each item. The measured children’s centre nutrition practices variables were assessed using items adapted from published scales that assessed the nutrition environment of childcare centres and kindergartens (Table 1) [45, 46].

### Statistical analyses

The distribution of all measured variables was screened prior to modelling. Two variables, the healthy and unhealthy food environment scores, were positively skewed and subsequently log transformed. To set a common scale for the analyses, all variables were standardised to have a mean of zero and standard deviation of one. Stata statistical software package version 13.0 [47] was used to transform variables, conduct sensitivity analyses (t-test for continuous variables and Chi Squared test for categorical variables) and to summarize participants’ socio-demographic, behavioural, psychological and environmental variables. IBM SPSS Statistics 22.0 [48] was used for exploratory factor analysis and IBM SPSS AMOS 22.0 [49] for model testing.

Testing a model in SEM involves two key stages: i) **measurement model** – confirms measured variables are significantly and adequately related to the model’s constructs; and ii) **structural model** – tests validity of relationships between constructs in the model [20]. Confirmatory factor analysis was applied to validate three measurement models incorporating: i) dietary behaviours, psychological, perceived affordability and perceived accessibility latent constructs, ii) spatial access to food outlets construct, and iii) children’s centre nutrition practices construct. For the structural model, given that the three environmental constructs are likely to be related, covariance between these three constructs was set. The environment of main supermarket construct was defined by a single measured variable. It is recommended in these cases that the error variance be set to the variance multiplied by [s.d.^2^ * (1-α)] [50], where α is the Cronbach’s alpha statistic.

The measurement and structural models were assessed for fit using five fit indices: Goodness-of-Fit Index (GFI ≥0.90 indicates good fit), Adjusted Goodness-of-Fit Index (AGFI ≥0.90 indicates good fit), Comparative Fit Index (CFI ≥0.90 indicates good fit), Root Mean Square Error of Approximation (RMSEA < 0.08 indicates good fit), and standardised Root Mean Square Residual (sRMR < 0.08 indicates good fit) [20, 51]. Bootstrap procedure is recommended for testing the significance of indirect effects [52]. It was run with 2000 samples to produce pathway coefficients and 95% bias-corrected confidence intervals for the overall indirect associations of each environmental construct on diet through the three psychological constructs because AMOS is unable to examine the indirect effects of specific pathways containing latent constructs. In the adjusted model, confounding variables (age, number of children, educational attainment and quintile of neighbourhood deprivation) were added by creating direct associations from each covariate to the dietary behaviour latent construct and the psychological constructs.

## Results

### Participants’ characteristics

Of the 921 women who completed the phase II SIH survey, 82% (*n* = 753) had complete data and were included in this study. Participants excluded (*n* = 168), due to incomplete data, had higher educational attainment (*p* < 0.001) than those included but showed no difference in age, number of children or level of neighbourhood deprivation (*p* > 0.2).

Table 2 presents the socio-demographic characteristics and dietary behaviours of the 753 participants with complete data. The mean age of the sample was 32 years (SD 6) and the vast majority had one or two children (80%). The age of children ranged from new born to 17 years but over three quarters (77%) were aged 5 years or younger. More than one third of participants (37%) had not attained an educational qualification after the age of 16 years and nearly a quarter (22%) lived in neighbourhoods within the most deprived quintile in England. Almost two thirds (60%) reported not being in paid employment. The mean dietary quality score for the sample was zero (SD 1), and the scores ranged from − 2.8 to 2.9. One standard deviation improvement in dietary score is equivalent to eating salad vegetables up to six times more often, and crisps up to six times less often a week. More than two thirds of women (70%) reported eating fruit once a day or more, while 38% and 21% reported never eating fast food or takeaway in the past month respectively.

**Table 2:** Sociodemographic characteristics and dietary behaviours of the participant sample (*n* = 753)

Mother’s characteristic	Mean	Standard Deviation (SD)
Age at interview	32	6
Dietary quality score	0.0	1
	n	%^a^
Number of children		
Pregnant	5	1
1	299	40
2	302	40
3	109	14
4+	38	5
Educational attainment		
Low (≤16 years of age)	282	37
Mid	276	37
High (degree)	195	26
Neighbourhood deprivation		
Most deprived	164	22
2	159	21
3	218	29
4	107	14
Least deprived	105	14
Fruit intake		
Never	18	2
Once a month	7	1
Once a fortnight	11	1
1–2 times a week	64	9
3–6 times a week	129	17
Once a day	275	37
> once a day	249	33
Fast food intake		
Never	286	38
Once a month	223	30
Once a fortnight	147	20
1–2 times a week	92	12
3–6 times a week	4	1
Once a day	1	0
> once a day	0	0
Takeaway food intake		
Never	155	21
Once a month	229	30
Once a fortnight	202	27
1–2 times a week	159	21
3–6 times a week	8	1
Once a day	0	0
> once a day	0	0

^a^Percentages may not add up to 100% due to rounding

Table 3 presents the descriptive findings for each of the psychological and environmental measured variables grouped by construct. The average for all psychological resources variables was towards the upper limit of the scales indicating women generally felt a good sense of control over life, confidence in eating healthily and health benefits resulted from eating healthily, and were involved in food-related activities. Not having enough money to buy food or balanced meals was a problem for less than a fifth of women (17% and 13% respectively). Most women agreed or strongly agreed that they could complete their food shopping (75%), had a good variety of fruit and vegetables (67%), and had good quality fresh produce (79%) in their local neighbourhood. The spatial access to food outlet measures showed that the median geographical area of women’s individualised activity spaces was 10 km^2^ (IQR 8, 12) and that most women had access to three different types of supermarkets within this area. The median food environment score for healthy outlets was 98SD (IQR 69, 136) and the median food environment score for unhealthy outlets was -175SD (IQR -122, − 615), indicating that most women had greater exposure to unhealthy than healthy food outlets while undertaking their daily activities. The median healthfulness score for the 51 supermarkets where participant’s purchased most of their groceries was 1.8SD (IQR 1.7, 1.9), with scores ranging from − 0.7 to 2.2. One-SD difference in healthfulness score is equivalent to a more healthful store having 11 more varieties of healthy foods, double the number of healthier alternatives of less healthy food products, and a cheaper mean price (£/portion) of the healthy than the less healthy foods (up to 31 pence). The children’s centre nutrition practice measures showed a high median food policy score (40SD; IQR 33, 41), healthy eating ethos score (40SD; IQR 37, 41) and healthy eating information score (9SD; IQR 8, 10), indicating that promoting healthy eating was a priority in most centres; though staff reported a moderate number of barriers to promoting healthy eating (4SD; IQR 3, 4).

**Table 3:** Individual and environmental variables for the participant sample (*n* = 753)

Construct Measured variable	Mean (SD)	Range
Psychological resources		
Perceived control over life	26 (2)	16 to 36
Self-efficacy for healthy eating	14 (2)	6 to 20
Healthy eating outcome expectancies	18 (6)	6 to 24
Food involvement	44 (5)	29 to 59
	*n*	%^a^
Perceived food affordability		
Can’t afford enough food		
Never	627	83
Sometime	103	14
Often	23	3
Can’t afford balanced meals		
Never	658	87
Sometime	72	10
Often	23	3
Perceived food accessibility		
Can food shop locally		
Strongly disagree	31	4
Disagree	158	21
Agree	400	53
Strongly agree	164	22
Limited variety of fresh F&V locally		
Strongly disagree	103	14
Disagree	401	53
Agree	223	30
Strongly agree	26	3
Good quality produce locally		
Strongly disagree	12	2
Disagree	142	19
Agree	522	69
Strongly agree	77	10
	Median (IQR)	Range
Spatial access to food outlets		
Area (km2) of activity space	10 (8, 12)	4 to 18
Supermarket variety	3 (3, 4)	2 to 4
Food environment score – healthy outlets	98 (69, 136)	12 to 445
Food environment score – unhealthy outlets	−175 (−122, −615)	−9 to − 810
Environment of main supermarket		
Healthfulness score	1.8 (1.7, 1.9)	−0.7 to 2.2
Children’s centre nutrition practices		
Food policy content	40 (33, 41)	16 to 43
Healthy eating ethos	40 (37, 41)	33 to 45
Healthy eating information	9 (8, 10)	6 to 11
Barriers to promoting healthy eating	4 (3, 4)	1 to 7

### SEM

Model fit was good for the dietary measurement model (GFI = 1.00, AGFI = 0.97, CFI = 0.97, RMSEA = 0.06 [0.02, 0.11], sRMR = 0.02) and for the measurement model incorporating the three psychological constructs (GFI = 0.98, AGFI = 0.96, CFI = 0.96, RMSEA = 0.05 [0.04, 0.07], sRMR = 0.04). The spatial access to food outlets measurement model showed adequate fit (GFI = 0.99, AGFI = 0.96, CFI = 0.99, RMSEA = 0.09 [0.05, 0.13], sRMR = 0.01). The children’s centre nutrition practices nutrition model revealed good fit for all indices (GFI = 0.98, AGFI = 0.92, CFI = 0.97, sRMR = 0.04), except RMSEA (0.12 [0.08, 0.17]). Post hoc modifications showed that placing a co-variance between the error terms for the healthy eating ethos and food policy variables improved model fit (GFI = 1.00, AGFI = 0.98, CFI = 1.00, RMSEA = 0.06 [0.00, 0.13], sRMR = 0.01). This action is justified by evidence showing that organisational policies influence organisational ethos, particularly the behaviours and attitudes of management and staff [53].

Overall model fit for the structural model was good (GFI = 0.93, AGFI = 0.91, CFI = 0.91, RMSEA = 0.05 [0.05, 0.06], sRMR = 0.05). Figure 1 shows that the indicator variables generally loaded well on the latent constructs with most (72%) having factor loadings greater than 0.50 and only one a poor factor loading (< 0.32). All measured variables showed significant associations with their corresponding construct (*p* < 0.01) and the total variance in diet explained by the psychological and environmental constructs was 37%.

### Associations

**Fig. 2:**
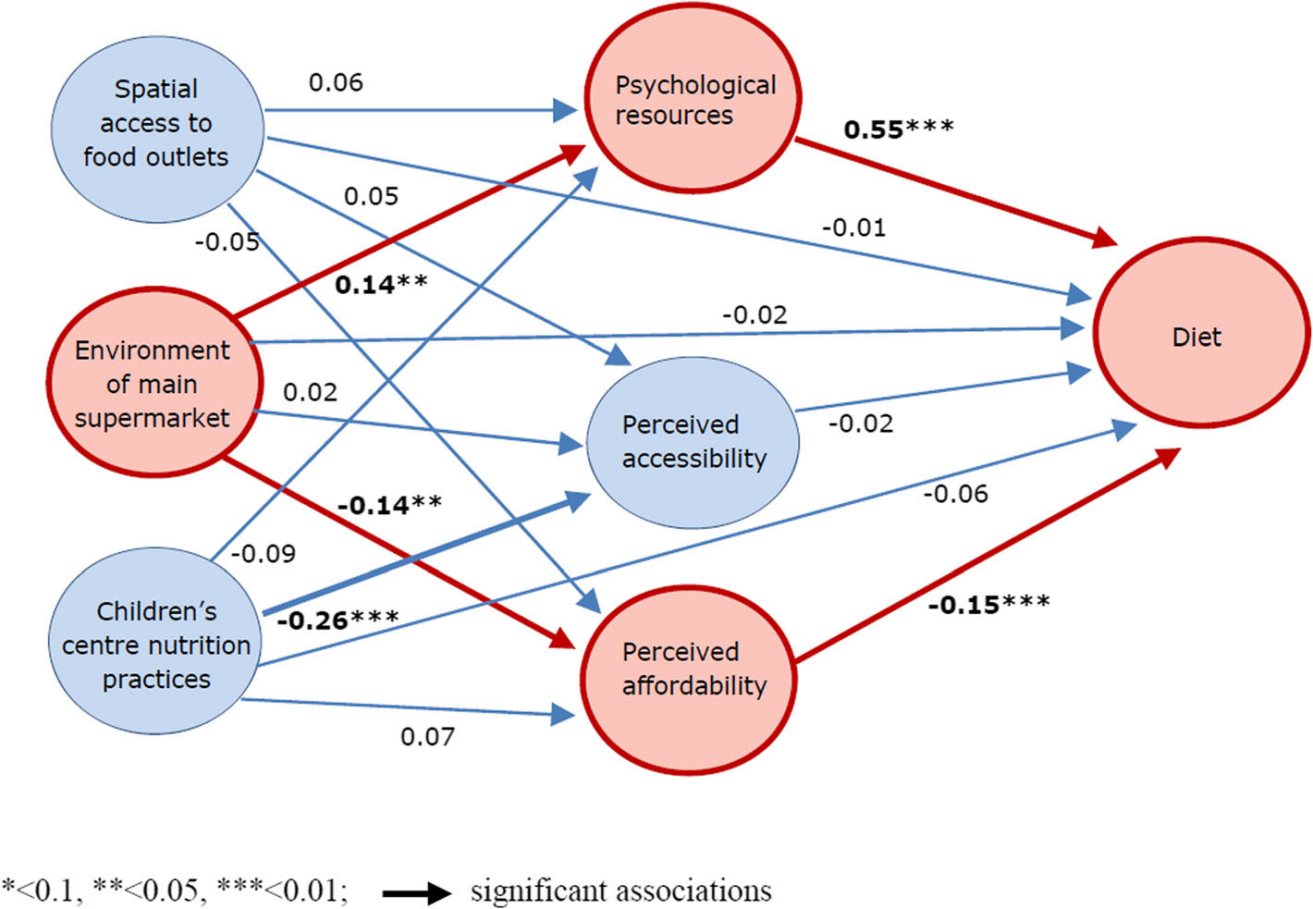
Structural model showing standardised regression weights between constructs

Figure 2 shows the standardised regression weights of associations between constructs for the structural model. None of the environmental constructs were directly associated with diet (all *p* > 0.3). Spatial access to food outlets was not associated with any of the psychological constructs (all *p* > 0.2) and children’s centre nutrition practices was only associated with one psychological construct (β = − 0.26SD, *p* < 0.001; others *p* > 0.1): better centre nutrition practices were associated with poorer perceptions of local healthy food access. Perceived access to healthy food however was not associated with the diet construct (*p* = 0.7). The environment of women’s main supermarket was positively associated with the psychological resources construct (β = 0.14SD, *p* = 0.03) and negatively associated with the perceived food affordability construct (β = − 0.14SD, *p* = 0.01), whereby women who shopped at supermarkets with healthier environments (i.e. better availability, pricing and placement of healthy foods) had more psychological resources attuned to healthy eating and fewer concerns about affording food or balanced meals. The psychological resources and perceived food affordability constructs were both significantly associated with the diet construct: women with more psychological resources attuned to healthy eating had better diets (β = 0.55SD, *p* < 0.001), while those with greater food affordability concerns had poorer diets (β = − 0.15SD, p = 0.01). The indirect association between supermarket environment and diet through the three psychological constructs was significant (β = 0.07, 95%CI 0.004, 0.071). Indirect associations with diet through the three psychological constructs were not significant for spatial access to food outlets or children’s centre nutrition environment (β = 0.03, 95%CI -0.01, 0.07 and β = − 0.04, 95%CI -0.08, 0.01 respectively).

Adjustment for covariates known to predict diet (age, number of children, educational attainment, neighbourhood deprivation) weakened the model goodness of fit indices slightly (GFI = 0.92, AGFI = 0.89, CFI = 0.89) however, the badness of fit indices remained within recognised limits (RMSEA = 0.06 [0.05, 0.06], sRMR = 0.06). There was little alteration in the strength or significance of the associations between constructs after adjustment; only the relationship between perceived food affordability and diet attenuated (*p* = 0.2). Each of the four potentially confounding variables were significantly associated with the diet construct (all *p* < 0.05). The indirect association between main supermarket environment and diet, through the three psychological constructs, remained significant after adjustment (β = 0.03, 95%CI 0.004, 0.07).

## Discussion

This study is one of very few to examine the pathways between dietary behaviour and multiple food environment and psychological factors. The overall model predicted dietary behaviours well. The results support the hypothesis that low-agency environmental determinants and high-agency individual determinants are synergistically associated with dietary behaviours: the in-store environments of women’s primary supermarkets were indirectly associated with their dietary behaviours, acting via their individual-level resources. More specifically, shopping at less healthful supermarkets (where availability, pricing and promotion favoured unhealthy foods) was associated with women having fewer psychological resources for healthy eating and poorer dietary behaviours. Use of less healthful supermarkets was also associated with greater food affordability concerns and poorer dietary behaviours (though the latter weakened after adjustment for confounding variables). Our results showed that the association between psychological resources and dietary behaviour had the largest effect size of all associations in the model. However, the strength of relationships between the supermarket environment and psychological resources and food affordability resources were not insubstantial, particularly if population reach is considered [54]. No direct associations between women’s dietary behaviours and the three environmental factors or women’s perceived access to healthy food access were observed.

This pathway analysis pinpoints three focal points for intervention to improve population diet: i) the in-store environment of supermarkets, ii) an individual’s psychological resources for healthy eating and iii) an individual’s perceived affordability of healthy food. Moreover, our findings suggests that interventions are most likely to be effective if strategies targeting these focal points are implemented concurrently. Supermarkets are an important source of food for many people [55–57] and therefore offer an important setting for public health intervention to improve population diet. A systematic review of nutrition intervention studies in supermarkets and grocery stores revealed good evidence that low-agency price reduction interventions increase purchases and/or intake of healthy foods, and some evidence for high-agency nutrition information strategies (e.g. shelf or product labels, posters and flyers) improving dietary behaviours [58]. Assessment of study quality however identified that most of the research was poor, having medium to high risk of bias. Three high quality randomised control trials from New Zealand [59], Australia [60] and the Netherlands [61] assessed the independent effects of: i) nutrition/behaviour change materials, ii) price reduction (12.5, 20 and 50% respectively) on fruit and vegetables, iii) price reduction plus nutrition/behaviour change materials or iv) no intervention, on supermarket purchases of targeted foods. The mechanisms underlying these interventions were that increased psychological resources (i.e. nutrition knowledge, self-efficacy) in addition to price reductions on healthy foods would together improve the healthiness of food purchases. These three studies showed that nutrition/behaviour change materials alone had no effect on fruit and vegetable purchases while price reduction alone and price reduction plus nutrition/behaviour change materials increased fruit and vegetable purchases; though the latter had little additive effect over price reduction alone. At first glance, these effectiveness trials appear inconsistent with our modelling results. However, the nature of the price reduction strategies meant that participants were not blinded to the intervention and were fully aware of the reduced cost of fruit and vegetables. It is therefore highly probable that participants’ perceptions of the affordability of healthy foods (i.e. fruit and vegetables) improved, particularly among low-income groups; whether this mediated the increase in purchasing was not tested in these studies.

The process evaluation results from the Australian trial supports the notion that price reduction improved perceptions of fruit and vegetable affordability [62]. More than two thirds of the participants who used the price discounts reported doing so because they saved money. Additionally, many perceived that the discount enabled them to buy more fruit and vegetables, or a greater variety of fruit and vegetables, particularly the more expensive types. Furthermore, participants reported that the discount made them feel appreciated and rewarded for making healthy choices; suggesting that low-agency environmental strategies may also enhance an individual’s psychological resources. This process evaluation, and our study results, suggest that strategies to improve the supermarket environment could help to improve an individual’s psychological resources. Something as obvious as product price reduction may facilitate improvements in self-efficacy and sense of control when buying healthy food products because individuals face fewer financial or physical barriers to healthy eating. Such mechanistic pathways are yet to be tested in intervention research but would be particularly relevant among low socioeconomic populations who hold fewer psychological resources than those more advantaged [63, 64].

Our findings suggest that high-agency interventions targeting individual psychological resources when combined with low-agency supermarket environment interventions may confer greater benefits on dietary behaviours than either intervention alone. The three supermarket trials described above found no such additive effect. One possible explanation is poor engagement with the nutrition/ behaviour change materials [61, 62]. Our measure of psychological resources excluded nutrition knowledge because there is growing consensus that just giving people information about what they need to do to change their health behaviour is ineffective [2]. By illustration, the Australian trial’s process evaluation revealed that participants could recall the healthy eating messages they received and reported liking the recipe ideas but used them infrequently [62]. An alternative to traditional educational approaches is to treat people, not as lacking knowledge, but as experts of their lives and their behaviours, helping them to break down their behaviours at the time and places where they occur and supporting them to act differently [2, 22]. Such an approach, when combined with low-agency environmental strategies, may help individuals to break the automatic patterns of purchasing unhealthy foods in some environments and create consistently healthy dietary practices. New technologies offer great potential to prompt people to reflect at times and places where they are undertaking dietary behaviours such as food shopping and cooking.

The lack of association we found between women’s dietary behaviours and their spatial access to food outlets or perceived access to healthy foods is consistent with findings from a systematic review of observational food environment research [17]. A systematic review of intervention studies measuring the effect of a new supermarket opening on the diets of nearby residents also found little evidence that enhanced access to supermarkets improved dietary behaviours [16]. However, in contrast to our results, the review showed that perceived access to healthy foods improved consistently across studies and the authors recommended longer follow-up periods to ascertain possible delayed dietary effects. Another of our findings showed that better children’s centre nutrition environments were associated with poorer perceptions of healthy food access. We reason that children’s centres with good nutrition policies and healthy eating activities may heighten women’s awareness of the importance of eating healthily and of the high numbers of unhealthy food outlets in their neighbourhoods. The children’s centres in this study were predominantly located in more deprived neighbourhoods which have a high prevalence of fast food outlets [15]. Our work provides evidence to support continuation of nutrition-related activities in children’s centres.

### Strengths and limitations

In this study we used a novel application of SEM to determine the relative strength of associations in a multi-component model to pinpoint areas for future intervention to improve population diet. The model was derived from previous theoretical and empirical work. By using latent constructs, SEM enables relationships to be measured free of error because the error for each construct is estimated and removed, leaving only common variance to calculate more accurate relationship estimates [20]. Additionally, SEM enables simultaneous assessment of direct and indirect associations between multiple constructs allowing relative comparison of the strength of relationships in addition to providing measures of statistical significance [65]. The use of actual exposures, including main supermarket and activity spaces, and the temporal connection between the collection of food environment, individual and dietary data increases confidence in the study findings. Finally, our sample had good representation of individuals from disadvantaged backgrounds.

The findings of this study are limited by the use of cross-sectional data which precludes conclusions relating to cause and effect. The setting within Hampshire, UK, somewhat limits the generalisability of the findings to other populations. Our study showed good model fit, however, this does not necessarily conclude that all necessary constructs have been included in the model [65]. For example, the home nutrition environment and social support for healthy eating have been shown to play a significant role in path analyses of previous obesity-related pathway models [50, 66]. We did not account for potential self-selection bias in our analyses [67]. It is therefore not clear the extent to which women in our study chose to conduct their daily activities in the areas they did because of the food outlets available to them, and how this may have affected our results. We applied a model that was linear in direction which may have under-represented the interplay or antagonistic actions between constructs. Testing the model’s pathways in intervention studies among different populations could help validate and/or improve the model and enhance its generalizability.

## Conclusion

Our findings provide empirical evidence for individual dietary behaviours being linked to both the environments of the supermarkets where women shop and their psychological resources. Policy initiatives in supermarkets that are likely to be effective at improving population diet, including the provision of greater varieties and cheaper pricing of healthy foods whilst simultaneously reducing promotions of unhealthy foods. When coupled with interventions to enhance psychological resources, such as nutrition self-efficacy and perceptions of healthy food affordability, these strategies are likely to be maximally effective. Individual strategies that hold great potential, particularly among those with the poorest diets, are those that steer away from simply providing nutrition information and towards encouraging people to recognise environmental manipulations and to feel good about having made healthy food choices. Researchers have a vital role in working with retailers to scientifically evaluate, using factorial methodologies, the pathways identified in this study and any differential effects by socioeconomic status.
